# Kinetics of anti-SARS-CoV-2 IgG in saliva and serum post infection, and subsequent vaccination in South Africa

**DOI:** 10.4102/ajlm.v15i1.3052

**Published:** 2026-06-26

**Authors:** Maemu P. Gededzha, Mixo Sibiya, Celine Pellaton, Kubashni Woeber, Duduzile Nsibande, Nobuhle Mchunu, Brodie Daniels, Terusha Chetty, Reshmi Dassaye, Khanya Mohlabi, Shameem Jaumdally, Keertan Dheda, Ruth Lekalakala, Shabir A. Madhi, Elizabeth Mayne, Glenda Gray, Yves Levy, Craig Fenwick, Song Ding, Penny L. Moore, Ameena Goga

**Affiliations:** 1 Department of Virology, School of Medicine, Sefako Makgatho Health Sciences University, Pretoria, South Africa; 2 Department of Immunology, National Health Laboratory Service, Johannesburg, South Africa; 3 Service of Immunology and Allergy Unit, University of Lausanne, Lausanne University Hospital, Lausanne, Switzerland; 4 HIV and Other Infectious Diseases Research Unit, South African Medical Research Council, Cape Town, South Africa; 5 Biostatistics Research Unit, South African Medical Research Council, Durban, South Africa; 6 Centre for Lung Infection and Immunity, Division of Pulmonology, Department of Medicine, Faculty of Health Sciences, University of Cape Town, Cape Town, South Africa; 7 South African MRC Centre for the Study of Antimicrobial Resistance, University of Cape Town, Cape Town, South Africa; 8 Faculty of Infectious and Tropical Diseases, Department of Infection Biology, London School of Hygiene and Tropical Medicine, London, United Kingdom; 9 Department of Pathology, Faculty of Health Sciences, University of Limpopo, Polokwane, South Africa; 10 Medical Microbiology, Polokwane Laboratory, National Health Laboratory Service, Polokwane, South Africa; 11 South African Medical Research Council: Wits Vaccines and Infectious Diseases Analytics Research Unit, Faculty of Health Sciences, University of the Witwatersrand, Johannesburg, South Africa; 12 Wits Infectious Diseases and Oncology Research Institute, Faculty of Health Sciences, University of the Witwatersrand, Johannesburg, South Africa; 13 Division of Immunology, Department of Pathology, Faculty of Health Sciences, University of Cape Town, Cape Town, South Africa; 14 National Health Laboratory Service, Groote Schuur Hospital, Cape Town, South Africa; 15 The Vaccine Research Institute, Faculty of Médicine, Université Paris-Est Créteil, Créteil, France; 16 Department of Immunology, Henri-Mondor Albert-Chenevier Hospital, Créteil, France; 17 EuroVacc Foundation, Lausanne, Switzerland; 18 SAMRC Antibody Immunity Research Unit, School of Pathology, Faculty of Health Sciences, University of the Witwatersrand, Johannesburg, South Africa; 19 National Institute for Communicable Diseases, National Health Laboratory Service, Johannesburg, South Africa; 20 Centre for the AIDS Programme of Research in South Africa, Durban, South Africa; 21 Department of Paediatrics and Child Health, Faculty of Health Sciences, University of Pretoria, Pretoria, South Africa

**Keywords:** SARS-CoV-2, anti-S IgG, anti-N IgG, saliva, vaccination, breakthrough infection

## Abstract

**Background:**

Understanding mucosal immune response to Severe acute respiratory syndrome coronavirus 2 (SARS-CoV-2) infection and vaccination, and its correlation to systemic responses could improve vaccine development. There is limited data on mucosal and systemic antibody responses to SARS-CoV-2 infection and vaccination in African populations.

**Objective:**

This study assessed serum and saliva antibody ratios in a cohort of SARS-CoV-2 infected and vaccinated participants followed up for 12 months.

**Methods:**

This longitudinal study comprised of 67 SARS-CoV-2, PCR-confirmed participants who were recruited as part of a COVID-19 Point of Care Study, between January 2021 and September 2022. For this analysis, 254 serum and 214 saliva samples (205 of which were matched), were collected at baseline, 6, 9, and 12 months. Antibodies against Spike (S) and nucleocapsid (N) SARS-CoV-2 were assessed by in-house Luminex assay.

**Results:**

Serum and saliva anti-S IgG from were detected for up to 12 months in majority of participants (93.5% and 90%), regardless of vaccination status. There was a concordance between paired serum and saliva antibody in 80.5% anti-S IgG and 41.7% anti-N IgG of samples. There was a weak but significant correlation between the matched serum and saliva samples (*ρ* = 0.42, *p* < 0.001 for anti-S and *ρ* = 0.33, *p* < 0.001 for anti-N). Longitudinal analysis in participants who were vaccinated with either the Ad26.COV2 or BNT162b2 COVID-19 vaccine after their baseline visit, serum anti-S IgG significantly increased (*p* = 0.023 and *p* = 0.038), compared with unvaccinated participants. Breakthrough infections or reinfections were identified in (35/62) 56.5% of vaccinated and unvaccinated participants.

**Conclusion:**

This study reports the correlation of the anti-S and anti-N IgG responses in serum and saliva following SARS-CoV-2 infection and subsequent vaccination in low-middle income setting. Furthermore, high breakthroughs and reinfection were reported.

**What this study adds:**

The study findings are consistent with those from high-income settings, supporting correlation between systemic and mucosal immunity and booster vaccination as a long-term strategy to control SARS-CoV-2.

## Introduction

South Africa, a middle-income country with high HIV prevalence was affected severely by the coronavirus disease 2019 (COVID-19) pandemic,^1^ and has experienced low uptake of Severe acute respiratory syndrome coronavirus 2 (SARS-CoV-2) vaccination. Despite the roll-out and effectiveness of COVID-19 vaccines in many countries, uncertainties remain regarding their long-term protection.^2^ SARS-CoV-2 infection induces humoral and cellular immune responses.^3,4^ Humoral immune responses include immunoglobulins (Ig) targeting SARS-CoV-2 proteins, especially the Spike (S) following vaccination, and both S and nucleocapsid (N) proteins following natural infection.^5^ Of these responses, anti-spike antibodies may neutralise the virus-retarding transmission.^6^

IgG is the most abundant immunoglobulin in the serum, and detection of anti-SARS-CoV-2 IgG contributes to evaluating infection and vaccine-induced immune responses.^7,8^ Severe acute respiratory syndrome coronavirus 2 anti-S IgG persists for several months after infection and correlates strongly with neutralising antibody activity.^9,10^ Both binding and neutralising antibodies have been associated with protection against different SARS-CoV-2 variants.^11,12^ Nevertheless, a recent population study highlighted that systemic antibody responses only account for a moderate proportion of protection against infection, suggesting that other immune responses may be better correlates of protection.^13^ SARS-CoV-2 serum antibody titres wane with time, with consequent decreased protection from infection and severe disease.^13,14^ Peripheral blood antibody responses may not reflect mucosal (nasal mucus and salivary) antibody responses, which may reflect better immunological protection.^13,15^ Mucosal neutralising antibodies are correlated with the protective efficacy of SARS-CoV-2 infection.^16^ Studies exploring the differences in mucosal and systemic antibody responses after SARS-CoV-2 infection, vaccination and infection mixed with vaccination, reported variability in mucosal antibody levels and durability, influenced by the type of exposure.^17,18,19,20,21,22,23^

Salivary IgG peaks within a month, persists for up to 9 months after SARS-CoV-2 infection,^17,24^ and can persist for 15 months after vaccination.^25^ Moreover, mucosal vaccination, which would more closely resemble natural infection, may produce more durable mucosal immunity than vaccines given intramuscularly.^13,26^ There are limited data on mucosal and systemic antibody responses to SARS-CoV-2 infection and vaccination in African populations. Therefore, understanding the mucosal immune response to SARS-CoV-2 infection and vaccination, and its correlation to systemic responses, is important to inform vaccine development.

This study assessed the correlation between SARS-CoV-2 serum and saliva IgG responses after vaccination, breakthrough infections and reinfections in a cohort of SARS-CoV-2 infected and vaccinated participants followed up for 12 months.

## Methods

### Ethical considerations

Written informed consent was obtained from participants, which included: ascertaining demographic information, COVID-19 vaccination status, HIV status, symptoms and comorbidities, and to collect venous blood in serum separator tubes and saliva samples at different time points. This study was approved by the human research ethics committee of the South African Medical Research Council (EC 005-4/2020), University of Witwatersrand (M200656), University of Limpopo (TREC/67/2020) and University of Cape Town (387/2020). The participants were allocated a unique identification number and no information was released or published by which samples can be traced back to patients.

### Study design

This was a longitudinal study using available serum and saliva samples, including both paired and unpaired samples collected at different time points (baseline, 6 months, 9 months, and 12 months).

### Study population and setting

The study population comprised 67 volunteers who were recruited as part of the South African Medical Research Council (SAMRC) led COVID-19 Point of Care (POC) study.^27,28^ The POC study included symptomatic persons under investigation (PUI) with possible acute COVID-19, and asymptomatic contacts suspected of having acute COVID-19 who presented at health facilities in two urban sites in Gauteng and the Western Cape, and one peri-urban site in Limpopo, South Africa in the period January 2021 to September 2022. During this period, South Africa experienced multiple COVID-19 waves caused by distinct SARS-CoV-2 variants. The Beta variant (B.1.351) dominated from early 2021 to May 2021, the Delta variant (B.1.617.2) from May 2021 to September 2021, and the Omicron variant (B.1.1.529) from November 2021 to September 2022.

For this study, 254 serum samples and 214 saliva samples (205 of which were matched), were considered sufficient for exploration and comparative analyses. This approach is consistent with similar studies^17,19,21^ where sample numbers were limited.

Participants who tested positive for COVID-19 underwent the POC rapid tests,^28^ and were then tested routinely for SARS-CoV-2 with real-time quantitative polymerase chain reaction on nasal or oropharyngeal swabs using a GeneXpert SARS-CoV-2 (Cepheid, Sunnyvale, California, USA), ThermoFisher TaqPath assay (Thermo Fisher Scientific, Waltham, Massachusetts, USA), or Seegene Allplex SARS-CoV-2 assay (Seegene Inc., Seoul, South Korea) at the National Health Laboratory Service per routine national protocols. The assays were used for qualitative SARS-CoV-2 detection, and results were reported as positive or negative. The participants were recruited based on laboratory-confirmed SARS-CoV-2 infection, regardless of possible prior infection.

### Sample collection

The POC study enrolled symptomatic PUI with possible acute COVID-19, and asymptomatic contacts suspected of having acute COVID-19 presenting to health facilities in two urban sites in Gauteng, and Western Cape and one peri-urban site in Limpopo in South Africa between January 2021 and September 2022.

Venous blood (up to two tubes of venous blood in serum separator tubes) and 1 mL saliva samples were collected from participants at baseline 6 months, 9 months, and 12 months. The samples were transported in coolers with icepacks to the Clinical Laboratory Services (CLS) biorepository, Braamfontein. Serum tubes were centrifuged at 3500 rpm for 15 min on either a ROTINA 420R or 460R centrifuges (Andreas Hettich GmbH & Co. KG, Tuttlingen, Germany). Serum and saliva were stored at −80 °C in 200 µL aliquots.

### Laboratory analysis: Luminex serological binding assay

Antibodies against Spike (S) and nucleocapsid (N) SARS-CoV-2 were assessed by Luminex xMAP multiplexed bead-based technology,^29^ where S and N proteins were coupled to Luminex beads as described by the manufacturer (Bio-Plex amine coupling kit, Bio-Rad Laboratories, Hercules, California, USA). Briefly, 50 µL of the 1/300 diluted serum samples, together with negative and positive controls, were incubated with 1:100 S and N beads and read on a Luminex 200 (Bio-Rad Laboratories, Hercules, California, USA). In this semi-quantitative assay, the mean fluorescent intensity signals for the anti-S and anti-N IgG serum binding were expressed as ratios compared to a negative internal control of pooled pre-COVID-19 pandemic human serum. The mean fluorescent intensity signal (in relative fluorescence units) for each test serum sample was divided by the mean signal for the negative-control samples to yield a mean fluorescent intensity ratio that was used as normalised units or values between plates and the different Luminex instruments tested.^29^ Saliva samples (250 µL) were treated with 1 × phosphate-buffered saline (Sigma-Aldrich, St. Louis, Missouri, USA), 0.5% Triton X 100 (Sigma-Aldrich, St. Louis, Missouri, USA), and 0.02% sodium azide (Sigma-Aldrich, St. Louis, Missouri, USA). They were vortexed briefly, centrifuged at 14 000×g for 10 min at 4 °C and the 1/27 dilution supernatants, together with the negative control (phosphate-buffered saline), were assayed for anti-S and anti-N IgG, as described above.^29^

### Data analysis

Data were captured, cleaned and validated using Microsoft Access (Microsoft Office, 2021; Microsoft, Redmond Washington, USA). Continuous variables were tested for normality using the Shapiro-Wilk test. All showed departures from normality. The Spearman correlation (*ρ)* was used to assess correlations between saliva and serum ratios. Continuous variables were assessed using alpha and were thus presented as medians with interquartile ranges (IQRs). The Wilcoxon matched-pairs signed rank test was used to compare differences in antibody ratios in groups (i.e. within vaccine comparisons of different follow-up time points and saliva and serum comparisons within different follow-up time points). Categorical variables were summarised using frequencies and percentages. The Kruskal–Wallis test was used to assess the association between the re-infection and breakthrough infection individuals. A *p* ≤ 0.05 was considered significant. All statistical analyses were performed using GraphPad Prism 10 (GraphPad Software, San Diego, California, USA) and Stata software version 18 (Stata Statistical Software, StataCorp LLC, College Station, Texas, USA).

## Results

### Characteristics of the participants

The study included 67 participants, comprising 42 women with median ages of 48 years old, and 25 men with median ages of 45 years old. Symptoms were scored as mild (upper respiratory tract infections only) and moderate (lower respiratory tract symptoms, high fever or severe gastrointestinal symptoms).^30^ Most participants had mild-to-moderate COVID-19 symptoms, with both mild and moderate symptoms being slightly more common among men (40% and 48%) than women (26.2% and 40.5%). HIV positivity was self-reported only in women (11.9%), while seems an odd word to use here, seeing as everything refers to female patients comorbidities such as diabetes and hypertension were more common in women, with six individuals reporting both conditions. Only men reported having tuberculosis, asthma, and chronic obstructive pulmonary disease. Among the vaccinated participants (*n* = 31), the majority of the participants received the Pfizer BioNTech BNT162b2 (74.2%) as compared to the Johnson & Johnson Ad26.COV2 (25.8%) vaccine after their baseline visit. A significant portion remained unvaccinated throughout the study. All 67 participants had serum and saliva samples, of which 53 participants were paired at baseline, 45 participants at 6 months, 57 participants at 9 months, and 50 participants at 12 months (Table 1a and Table 1b).

**TABLE 1a:** Age median and interquartile range of participants in the study.

Variable	Female (*N* = 42)		Male (*N* = 25)	
	Median	IQR	Median	IQR
Age (years)	48	34–56	45	34–50

IQR, interquartile range.

**TABLE 1b:** Characteristics of participants included in the study.

Variable	Female (*N* = 42)		Male (*N* = 25)	
	*n*	%	*n*	%
**Disease severity** †				
Asymptomatic	11	26.2	2	8.0
Mild	11	26.2	10	40.0
Moderate	17	40.5	12	48.0
No data‡	3	7.1	1	4.0
Self-reported HIV positive status	5	11.9	0	0.0
**Self-reported comorbidities**				
Tuberculosis	0	0	2	8.0
Diabetes mellitus	6§	14.3	1	4.0
Hypertension	10§	23.8	3	12.0
Asthma	0	0	1	4.0
COPD	0	0	1	4.0
**Vaccination**				
Johnson and Johnson Ad26.COV2	7	17.0	1	4.0
Pfizer BioNTech BNT162b2¶	15	36.0	8	32.0
Vaccinated prior to enrolment††	3	7.0	2	8.0
Not vaccinated	17	40.0	14	56.0

COPD, chronic obstructive pulmonary disease.

† , Disease severity classified according to WHO COVID-19 clinical management guidance.^30^

‡ , Disease severity of the participants was not recorded.

§ , Six of the participants had diabetes mellitus and hypertension.

¶ , Twelve participants (8 women and 4 men) were partially vaccinated (i.e. received only one dose).

†† , Three participants were vaccinated with Pfizer and two with Johnson&Johnson.

### Baseline and longitudinal seroprevalence

Serum anti-S IgG was detected in 87.6% (95% confidence interval [CI], 77.1–94.5) at baseline, and in 96.6% (95% CI, 88.2–99.5), 98.4% (95% CI, 91.4–99.9) and 93.5% (95% CI, 84.2–98.2) at 6-months, 9-months, and 12-months time points. Salivary anti-S IgG was detected in 61.1% (95% CI, 46.8–74.1) of individuals at baseline, which showed a slight increase at 6-months (88.8%; 95% CI, 75.9–96.2), 9-months (86.8%; 95% CI, 75.7–94.1), and 12-months (90%; 95% CI, 78.2–96.6) time points.

Serum anti-N IgG was detected in 75.3% (95% CI, 63.1–85.2) at baseline, and in 81.3% (95% CI, 69.1–90.3), 79.3% (95% CI, 67.3–88.5) and 72.5% (95% CI, 59.7–83.1) at 6-month, 9-month, and 12-month time points. Salivary anti-N IgG was more variable and lower, detected in 31.4% (95% CI, [19.5–45.5]) of individuals at baseline, and gradually decreasing to 22.9% (95% CI, 12.0–37.3) by 12 months.

### Relationship of anti-S and anti-N responses in serum and saliva

**FIGURE 1:**
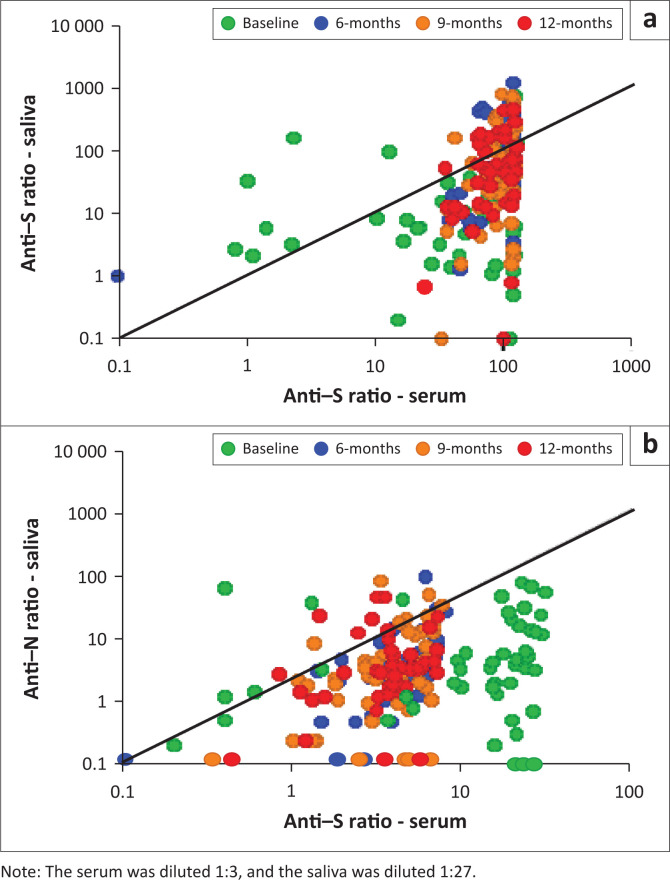
Correlation analysis of anti-S and anti-N IgG between (a) 205 serum and (b) 204 saliva samples across a 12-month follow-up period.

Pooled data showed that there was concordance at any of the time points between the matched serum and saliva IgG antibody ratios in 165/205 (80.5%) of anti-S IgG and 85/204 (41.7%) of anti-N IgG results. There was a moderate positive correlation (*ρ* = 0.42, *p* < 0.001 for anti-S) and a weak (*ρ* = 0.33, *p* < 0.001 for anti-N), but significant correlation between the matched serum and saliva samples (despite the fact that saturation (anti-S ratio of 120 and anti-N ratio of 25) was reached for many of the serum samples, for both anti-S and anti-N (Figure 1).

The POC study enrolled symptomatic PUI with possible acute COVID-19, and asymptomatic contact suspected of having acute COVID-19 presenting to health facilities in two urban sites in Gauteng and the Western Cape, and one peri-urban site in Limpopo in South Africa between January 2021 and September 2022.

### Longitudinal changes in serum and saliva ratios

**FIGURE 2:**
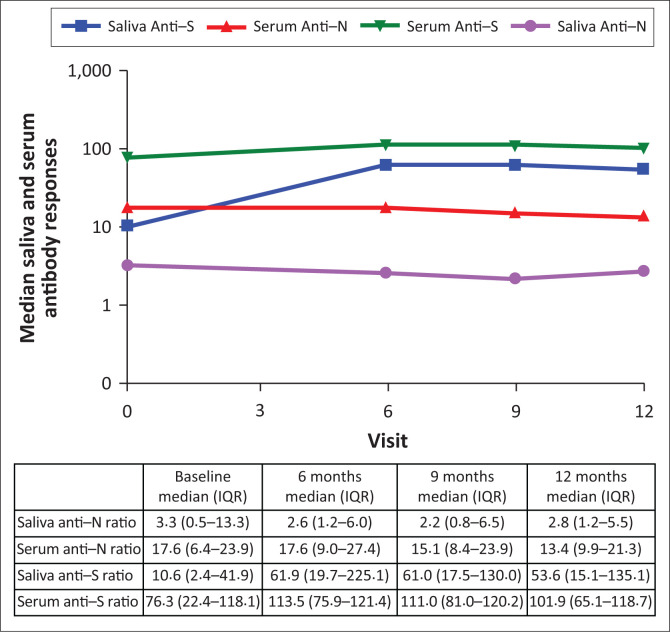
Longitudinal analysis of Anti-S and Anti-N ratios in serum and saliva across the participants at indicated time points.

The change in antibody ratios over time was quantified, by comparing IgG antibody ratios (relative to a negative control pool of pre-COVID-19 pandemic human serum) at four time points (baseline, 6, 9, and 12 months). The median ratio of anti-S IgG was higher compared with baseline at each of the subsequent time points in both the serum and saliva samples (Figure 2).

Serum anti-S ratios increased from a baseline median of 76.3 (IQR: 22.4–118.1) to 113.5 (IQR: 75.9–121.4) at 6 months (*p* = 0.003), remained stable at 9 months [111.0 (81.0–120.2); *p* = 0.934], and then declined to 101.9 (65.1–118.7) at 12 months (*p* = 0.049). In contrast, serum anti-N levels remained unchanged from baseline [17.6 (6.4–23.9)] to 6 months [17.6 (9.0–27.4)] (*p* = 0.424), but significantly decreased at 9 months [15.1 (8.4–23.9)] (*p* = 0.011) (see Figure 2).

### Correlation of antibody titres by comorbidities or symptom severity

The participants with moderate COVID-19 had higher anti-N serum ratios compared to asymptomatic individuals at 6 months (23.7 [12.9–28.3] vs 11.5 [7.8–15.8]; *p* = 0.0095), 9 months (17.1 [10.2–25.6] vs 8.6 [3.9–13.1]; *p* = 0.013) and 12 months (15.8 [10.2–24.2] vs 10.8 [5.5–12.3]; *p* = 0.004). The serum anti-N IgG ratios were higher for participants with comorbidities than for those without comorbidities at 6 months (22.7 [16.9–28.1] vs. 13.9 [8.8–24.0]; *p* = 0.042), 9 months (24.6 [14.7–26.8] vs. 10.7 [4.9–17.9]; *p* = 0.001), and 12 months (16.9 [12.1–25.9] vs. 10.7 [4.9–17.9]; *p* = 0.028).

### Antibody responses by vaccine

Longitudinal analysis for serum anti-S IgG ratios in participants vaccinated with BNT162b2 increased significantly from baseline (94.7 [27.4–118.4]), to 9 months (102.8 [88.6–120.0]), *p* = 0.038, while in the Ad26.COV2 participants, anti-S IgG ratios increased from baseline (71.8 [10.2–103.9]) to 6 months (119.7 [114.3–121.8]), *p* = 0.023. There was no statistical difference in the serum anti-S IgG ratios for the unvaccinated participants. The serum anti-S IgG ratios remained stable at 12 months for both Ad26.CoV2 and BNT162b2, while unvaccinated decreased slightly, although not significantly.

**FIGURE 3:**
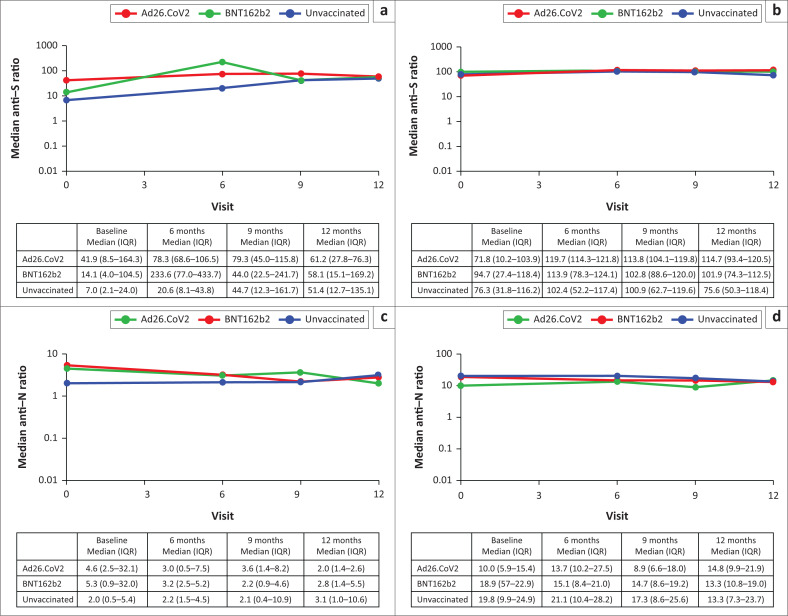
Longitudinal analysis of Anti-S and Anti-N ratios in serum and saliva according to vaccination status at different time points (Baseline (0), 6, 9, and 12 months). (a) Saliva Anti-IgG, (b) Serum Anti-S IgG, (c) Saliva Anti-N IgG and (d) Serum Anti-N IgG.

The saliva anti-S IgG ratios for participants vaccinated with BNT162b2 increased significantly, from baseline (14.1 [4.0–104.5]) to 6 months (233.6 [77.0–433.7]), *p* < 0.001, and decreased from 6 months (233.6 [77.0–433.7]) to 9 months (44.0 [22.5–241.7]), *p* = 0.008, and from 6 months (233.6 [77.0–433.7]) to 12 months (58.1 [15.1–169.2]), *p* = 0.008. Ad26.COV2 increased at different time points, albeit not significantly. However, the saliva anti-S IgG ratios of unvaccinated participants also increased significantly from baseline (7.0 [2.0–24.0]) to 9 months (44.7 [12.3–161.7]), *p* = 0.003. The saliva anti-S ratio for all three groups (Ad26.CoV2, BNT162b2, and unvaccinated) were at a similar level at 12 months. The saliva anti-N IgG ratios decreased significantly from baseline to 12 months in both Ad26.CoV2 (4.6 [2.5–32.1] vs. 2.0 [1.4–2.6], *p* = 0.031) and BNT162b2 (5.3 [0.9–332.0] vs. 2.8 [1.4–5.5], *p* = 0.027). There was no difference with the serum anti-N IgG ratios (Figure 3).

### Breakthrough infections or reinfections

**FIGURE 4:**
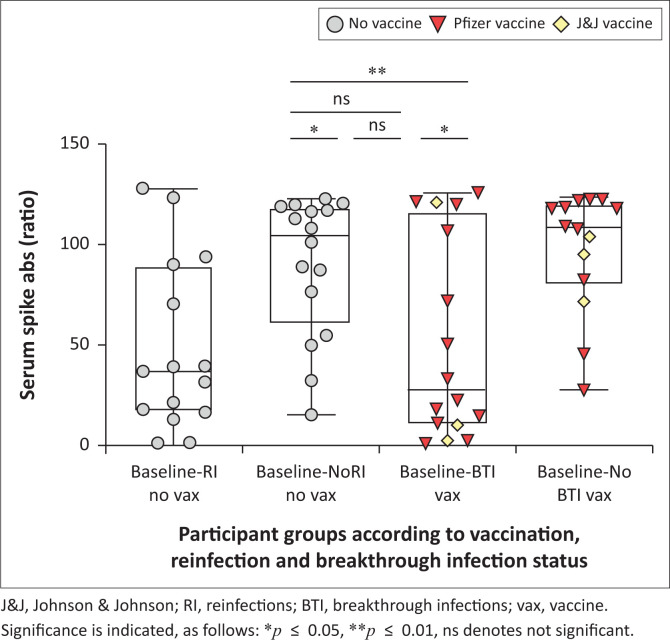
Baseline analysis of serum anti-S IgG comparing participants with re-infection and breakthrough in unvaccinated and vaccinated participants.

Breakthrough infections or reinfections were identified in 56.5% (35/62) of participants, based on a significant increase (> 2-fold, average increase of 3.84-fold) in serum anti-N IgG between time points, with 58% (18/31) of vaccinated participants having a breakthrough infection, and 54.8% (17/31) of unvaccinated participants having a reinfection. Breakthrough infections were identified in both the BNT162b2 (56.5%, 13/23) and Ad26.COV2 (75%, 6/8) groups between one to 12 months post vaccination. Participants with breakthrough infections (27.7 [10.2–118.4] and 108.1 [82.4–118.2]; *p* = 0.016) or reinfections (34.3 [15.9–79.9] and 104.5 [76.3–118.5]; *p* = 0.036) had lower serum anti-S IgG at baseline compared to those without (Figure 4). There was no statistical difference in the saliva anti-S IgG when comparing breakthrough infections or reinfections to those without (data not shown).

## Discussion

Assessing the robustness and persistence of antibody responses to infection and vaccination at the mucosal surface provides important data for understanding long-term immunity and vaccine efficacy.^31^ This paired saliva and serum study showed similar anti-S IgG responses in the mucosal and systemic compartments but significant differences in the anti-N IgG, following SARS-CoV-2 infection and vaccination. The overall prevalence of serum anti-S IgG and anti-N IgG was higher than that reported in a large South African seroepidemiological survey conducted from 22 October 2021 to 09 December 2021, consisting mainly of unvaccinated participants.^32^ Our study reported that saliva anti-S IgG ratios were detected for up to 12 months in the majority of participants, co-existing with serum ratios (90% vs. 93.5%). Persistent salivary IgG has been reported previously in natural SARS-CoV-2 infection^17^ and COVID-19 vaccination,^25^ supporting that saliva can be used as a matrix to assess mucosal immunity.

Despite the assay detecting anti-S and anti-N IgG in both saliva and serum, the data showed a weak, yet significant correlation between the serum and saliva anti-S and anti-N IgG across all time points. Although several studies have reported a significant correlation between serum and saliva,^18,33,34^ this study is in concordance with a previous study in the United Kingdom that reported a weak correlation among healthcare workers.^23^ The weak correlation in this study may be a result of lower levels of IgG in the saliva samples, especially in anti-N, as reported previously.^35^ There was a 1-log difference between the anti-S IgG in the saliva and the serum. Another study has reported a correlation despite saliva levels being approximately 3 log lower as compared to plasma levels.^36^ Similar to previous studies, anti-S IgG levels in this study were higher and persisted for a longer period than anti-N IgG.^35,37,38^ The detection of antibodies in the saliva, although with weak correlation, suggests that saliva can be used as an alternative specimen to assess immunity.^39^ Higher levels of anti-N IgG levels were reported in the mild and moderate COVID-19 and comorbid participants, and this is in agreement with previous studies.^40,41^

The study revealed a significant rise in the serum anti-S IgG levels following vaccination, supporting the ability to generate robust spike-specific antibodies in both Ad26.COV2 and BNT162b2 vaccines demonstrated in previous studies.^42,43^ Vaccine administration in previously SARS-CoV-2-infected participants has been shown to induce high levels of durable binding and neutralising antibody responses compared to uninfected participants, which might lead to enhanced systemic and mucosal antibody responses, protecting against progression to severe disease.^44,45,46^ However, care should be taken in interpreting these differences, since anti-S IgG levels were high and often at the upper detection limit of the Luminex assay under the experimental conditions. In addition, a study performed in South Africa reported a boost in anti-S IgG titres in Ad26.COV2.S-vaccinated South African patients with prior infection.^47^

This study is in agreement with previous studies, which have shown that saliva IgG antibodies in increase after BNT162b2 vaccination in participants with previous infection^45,46,48,49,50^ and natural infection,^45^ although at a low level, indicating an intense immune response in the mucosa which might lead to protection against SARS-COV-2 infection. Although Ad26.COV2 may be effective systemically, the modest increase in saliva IgG antibodies may reflect limited mucosal boosting capacity owing to a single dose.

This study also reported, in a cohort with prior SARS-CoV-2 infection, breakthrough infection in vaccinated and reinfection in unvaccinated participants, as measured by the significant increase in the anti-N serum IgG levels. Both vaccines target the spike protein,^51^ and the nucleocapsid is not included in the vaccines.^52^ The reported breakthrough infection rate (58%) in both vaccines is similar to a study in Pakistan that reported a 67% breakthrough infection rate in participants without prior infection,^53^ but higher than previously reported studies showing 13%^54^ and 3.9%.^55^ Similar to other studies, the low antibody levels in both serum and saliva samples prior to breakthrough infection have been reported previously.^56,57^ Low anti-S titres may serve as an indicator of poor prognosis in breakthrough cases,^58^ thus exposing the participants to a risk of reinfection. It is worth noting that the majority of participants developed breakthrough infections more than 3 months post vaccination. In addition, the majority of the breakthrough infections in this study occurred between November 2021 and May 2022, when the more transmissible omicron variants were circulating in South Africa.^59^ It has been reported previously that individuals infected with Omicron variants have a high rate of breakthrough and reinfections.^60,61^ A study by Moreira et al. highlighted the importance of the third dose of BNT162b2 vaccine in reducing infections.^62^ Breakthrough infections post-vaccination have been reported to boost neutralising antibodies and to contribute to high levels of immunity.^63^ The persistent duration of SARS-CoV-2 IgG in saliva and the increase in the number of participants positive for anti-S IgG levels over time suggest sustained mucosal immunity and potentially enhanced protection at mucosal surfaces.

### Limitations

Limitations of this study include the measurement of IgG responses only. It is possible that other types of antibodies, including anti-S mucosal IgA antibodies, may assess mucosal immunity better. Moreover, not all participants were vaccinated, and the severity of breakthrough or re-infections could not be assessed, thus reducing the ability to assess the effect of vaccination on subsequent infections authentically. The difference in the number of men and women is most likely because of volunteer participation, as women are generally more willing to participate in health-related research than men.

### Conclusion

In conclusion, this longitudinal study reports the correlation of the anti-S and anti-N IgG responses in serum and saliva following SARS-CoV-2 infection and subsequent vaccination. In addition, the level of protection from breakthrough and re-infections with SARS-CoV-2 appears to be related to some extent to antibody levels, and to the waning of IgG antibodies. This warrants vaccination and/or booster vaccination as a long-term strategy to control SARS-CoV-2, although the results of this study must be interpreted with caution because of the small sample size.
